# A real-world evaluation of the clinical benefits of improved sound processor technology among Chinese cochlear implant users: A focus on Cochlear Nucleus 7

**DOI:** 10.1371/journal.pone.0307044

**Published:** 2024-09-03

**Authors:** Xiaocong Deng, Chenjiong Wu, Lin Wu, Jiyun Lu, Jin Zhang

**Affiliations:** 1 Department of Head and Neck Surgery, Hainan Cancer Hospital, Haikou, Hainan, China; 2 Medical Affair Department, Cochlear Great China, Beijing, China; 3 Department of Otolaryngology and Head and Neck Surgery, Boao Super Hospital, Qionghai, China; Prince Sattam bin Abdulaziz University, SAUDI ARABIA

## Abstract

Real-world evidence is increasingly used to support clinical and regulatory decisions globally and may be a useful tool to study the unique needs of cochlear implant users in China. The ability to recognize and understand speech in noise is critical for cochlear implant users, however, this remains a challenge in everyday settings with fluctuating competing noise levels. The Cochlear™ Sound Processor, Nucleus^®^ 7 (CP1000), includes Forward Focus, a spatial noise algorithm aimed to improve speech-in-noise performance, and Made for iPhone/iPod/iPad functionality. We conducted a prospective, single-center, open-label, within-participant, real-world evidence investigation in participants with cochlear implants. The primary objective of this study, conducted in China, was to compare speech perception in spatially separated dynamic noise with the Nucleus 7 to the recipients’ current older Cochlear Sound Processor, including the Freedom and Nucleus 5 sound processors. A follow-up study monitored participants from the initial study up to 12-months post the fitting of their Nucleus 7 and investigated hearing ability, satisfaction, and usability of the device via a questionnaire. Forty participants were included in the initial study (age-range 3 to 49 years) and 29 continued to the follow-up study (age-range 5 to 28 years). The participants were heterogeneous in terms of age, cochlear implant experience, and duration of hearing loss. Nucleus 7 significantly improved participant speech recognition performance in noise by 7.54 dB when compared with the participants’ current older sound processor (p<0.0001). Overall satisfaction with Nucleus 7 was 72%. Satisfaction in different hearing contexts ranged from 93.1% for understanding a 1:1 conversation in a quiet setting, 62.1% for understanding on the phone, to 34.5% hearing in complex noisy situations. The study demonstrated the benefits of the Nucleus 7 sound processor across different hearing environments in a Chinese population and showed improved hearing ability, usability, and satisfaction in a real-world every-day environment.

## Introduction

Real-world evidence (RWE) is based on the collection of real-world effectiveness and safety data during routine clinical care [1]. Advantages of RWE compared with randomized clinical trials include the ability to track real-world patient and physician behavior, and inclusion of a broad population sample selected as suitable candidates for the intervention by their physician [1]. A further advantage of RWE in a country such as China, which accounts for more than 20% of the world’s population, is the ability to sample a broad population, of which more than 27.8 million have hearing loss. Such insights can contribute to understanding the potential benefits of intervention in the population to be treated [2]. RWE is increasingly being used to support clinical and regulatory decisions globally [1, 3, 4]. In the context of cochlear implant use, RWE can provide a more holistic view of user experience, focused not only on understanding speech in noisy environments, but also including other aspects of hearing such as appreciation of music and environmental sounds.

Understanding speech in noisy environments remains challenging for Cochlear Implant users [5]. The ability to recognize and understand speech in noise is critical, however, most real-world communication takes place in noisy environments [6]. Daily hearing conditions can vary enormously, with fluctuating competing noise levels commonly found in environments such as schools, workplace, family gatherings, or when participating in sport, or playing musical instruments. Noisy environments can have more profound impacts on children than adults, as children have less control over their environment than adults at times, with indoor noise levels in schools being higher than recommended maximum noise levels. This can lead to communication problems and negatively affect linguistic and cognitive development [5, 7]. Restricted speech recognition in noisy environments can have significant impacts on the well-being of children, including physical stress, fatigue, and feelings of isolation [5]. Conversely, in adults, older age is associated with poorer recognition of words in difficult sentences, suggesting that cognitive aging may also negatively impact cochlear implant users [8].

In the last three decades, since 1994 when the first cochlear implant surgeries occurred in mainland China [2], cochlear implant systems have incorporated a variety of sound processor innovations to external sound processors to enhance speech understanding in a range of real-world listening situations, leading to improved speech perception outcomes. Increasingly, lifestyle considerations and connectivity demands have become equally important as hearing performance to cochlear implant users [9–12]. Newer sound processor technology has been designed to be compatible with older generations of the internal components of the cochlear implant system, hence upgrading to the latest sound processor through “upgrading” does not require surgical intervention.

The evolution of sound processors has resulted in smaller, power efficient processors, with algorithms to enhance performance in a range of listening situations and connectivity [11]. The original body worn sound processors have made way for ear level or off the ear sound processors.

The Cochlear™ Sound Processor (SP), Nucleus^®^ 7 (CP1000), includes ForwardFocus, a spatial noise algorithm aimed to improve speech-in-noise performance, and Made for iPhone/iPod/iPad (MFI) functionality. At the time the study was conducted, Nucleus 7 was the only sound processor that could be directly connected to a smartphone for Bluetooth streaming and for device monitoring. The Nucleus Smart App is available for Nucleus 7 SP recipients as an optional accessory (mobile application), which can be downloaded onto compatible mobile devices and used via Bluetooth to monitor and control the CP1000 SP. Audio from mobile phones and other Apps can be directly streamed to the CP1000 SP.

Older generation SPs, such as Freedom, Esprit, Sprint, and Nucleus 5, in use in China do not include these advanced multiple microphone pre-processing technologies, such as direct streaming via Bluetooth. When speaking on the telephone, in the absence of a wireless link, cochlear implant users need to hold the phone to one ear and listen in a different mode from that used in everyday listening, which can limit understanding. Hence, wireless functionality may improve user experience and listening performance, particularly conversations over the phone, or in a noisy environment, and allow for appreciation of music [9].

Comparisons of newer cochlear implant SPs with their predecessors are important to confirm whether the incremental ergonomic and/or technical improvements result in real-world clinical benefits and user satisfaction [9]. However, there is limited real-world published data comparing the performance of cochlear implant users’ experience using their current older SP compared with more recent best-in-class technology.

In China, the Bo’ao Lecheng International Medical Tourism pilot area allows domestic patients to use or implant medical devices that are currently listed internationally but are not available in mainland China. This enables medical professionals and patients to gain early access and valuable experience with technologies and products. Moreover, the Hainan provincial government launched a pilot project to collect real-world applied clinical data in the Bo’ao Lecheng International Medical Tourism pilot area, with a view to generating RWE in typical Chinese populations [3].

We conducted a real-world study in the Boao Lecheng International Medical Tourism Pilot Zone. The primary objective of this real-world study was to compare speech perception in spatially separated dynamic noise with the Nucleus 7 SP (ForwardFocus enabled) (N7FF) to the recipients’ current older Cochlear Sound Processor, with their current preferred program. The secondary objective included comparing a participant’s subjective evaluation after using Nucleus 7 SP for up to 1 month, to the participants’ current older Cochlear SP experience, with their current preferred program. A follow-up study was also conducted to evaluate the satisfaction and usability of the Nucleus 7 SP and Nucleus Smart App via a questionnaire covering hearing ability, satisfaction, and usability after 12-months experience with the Nucleus 7 SP. The studies were conducted in everyday hearing environments among Mandarin speaking Chinese adults and children.

## Materials and methods

### Study design

The study was designed as a prospective, single-center, within-participant (same participant wearing current older SP and then Nucleus 7 for comparison), real-world evidence investigation. The study was open-label as, given the differences being evaluated, it could not be performed with a blinded method. The study design was a fixed sequence, cross-over design with a duration of 4 months from December 2019 to March 2020. The follow-up study evaluated participants from the initial study for up to 12-months post their Nucleus 7 SP fitting, from December 2020 to July 2021, and investigated the ongoing hearing ability, satisfaction, and usability of the Nucleus 7 SP.

Both studies were conducted in accordance with ethical principles that have their origin in the Declaration of Helsinki and Good Clinical Practice (GCP). The studies were approved by the Boao Super Hospital Ethics Committee and Local Provincial National Medical Products Administration (NMPA) of the Hainan Province (Reference # KY1912001 and # KY2009003). Ethics application considered the potential inclusion of vulnerable populations through clarifying inclusion and exclusion criteria to minimize any potential risk to participants. Study design had clear scientific goals with appropriate safeguards in place through comprehensive Informed Consent Forms (ICF) that were reviewed and approved by the ethics committee.

### Participants

All participants had received cochlear implants which is a routine procedure for the management of population with bilateral moderate sloping to profound sensorineural hearing loss in 12 months or older.

At the time of the study, the N7 sound processor was not available in mainland China, and could only be accessed in the Boao Medical Tourism zone. Participation was voluntary and inclusion was offered as part of the recipient’s decision to access the technological upgrade of their sound processor. Once fitted with the N7 sound processor the participants accessed their clinical services on the China mainland.

### Initial study

The inclusion criteria for the initial study included fluent Mandarin-speaking participants with cochlear implants with a speech test score in quiet at baseline ≥30% in the test ear (tested by the “HOPE” Computer-aided Mandarin Speech Test System with the target sentence presented at 60 dB sound pressure level (SPL) in the free field) and at least 12 months of use with their existing SP [13]. The inclusion criteria were implemented to ensure the participants had the capacity to undertake the language-based tasks. All participants were recipients of the Cochlear implant 24RE or later generations of Cochlear Nucleus implants. Written informed consent was obtained from the participants or from the parent/guardian of pediatric participants.

### Follow-up study

All participants in the initial study were invited to participate in the follow-up study after having at least 6 months of experience with the Nucleus 7 SP. Additional exclusion criteria to those already specified for the initial study included additional disabilities, as assessed by the investigator, that would prevent participation in evaluations and adults or parents/carers with limited Mandarin, as assessed by the investigator (that is, adults or parents/carers who spoke a dialect of Mandarin that would prevent completion of the questionnaires). The decision not to participate in the follow study did not impact the ongoing care on the China mainland.

### Variables

#### Initial study

The primary endpoint for the initial study was to evaluate the speech recognition threshold (SRT) in dynamic noise with N7FF compared to the participants’ current older SP, with their current preferred program. The capability of an individual listener to understand speech in noise is usually a measure of their SRT. The SRT is defined as the signal-to-noise ratio where the listener understands 50% of the speech material [14]. The speech signal was a male Mandarin speaker consisting of 20 sentences per list and 2 lists of sentences were measured per SP condition in spatially separated 4-talker babble noise presented from multiple directions. The SRT was conducted using the “HOPE” Computer-aided Mandarin Speech Test System with the target sentence presented at 60 dB SPL in the free field [13]. The secondary endpoint was the participant’s subjective gain in listening benefit in everyday environments after using Nucleus 7 SP for up to one month compared to the participants’ own current older SP, as assessed using an age-appropriate version of the Spatial, Speech and Quality of Hearing Scale (SSQ) questionnaire. The SSQ-12 is a standardized questionnaire devised to measure subjective performance in various listening situations with older children (aged over 10 years) and adults. The SSQ Pediatric, Parents edition (SSQ-P) [15] and SSQ-12 [16] were translated into Mandarin. Parents assisted the completion of the SSQ-P edition for children 10 years or younger.

#### Follow-up study

The primary endpoint of the follow-up study was to measure the participant’s self-reported hearing ability, satisfaction, and usability of the Nucleus 7 SP device after at least 6 months usage. The primary endpoint was evaluated using a 37-item questionnaire which was custom made to measure satisfaction and usability with the Nucleus 7 SP (questionnaire available in S1 File). An example of an item in the questionnaire was: ‘How satisfied are you or your child with your hearing performance in background noise?’ A 5-point Likert ranking scale was used for which five response options were provided: very satisfied, satisfied, neutral, dissatisfied and very dissatisfied. Response options were coded from 1 to 5, with high scores representing a high rank on satisfaction and usability. The questionnaire was developed as no validated measure of sound processor satisfaction and usability was readily available. The items were designed to portray four topics of functionality: (1) Hearing performance; (2) Functionality—telephone use; (3) Functionality—music and audio streaming; and (4) Functionality of the Nucleus Smart App.

### Statistical analysis

#### Initial study

The study was designed to detect superiority of the SRT score in dynamic noise for N7FF compared to the participants’ current older SP by using a paired t-test. A total sample size of 40 was planned based on a power of 0.8, a significance level α = 0.05 (two-tailed) and an expected standard deviation (SD) of difference in speech recognition domain in noise of 1.8. The SD was based on previous clinical trials comparing different sound processing methods using the HOPE adaptive sentence in noise test with Mandarin speaking cochlear implant recipients [13]. Based on the above assumptions, a sample size of 28 participants was needed. However, the aim was to enroll 40 participants to account for the possibility that variability in difference scores could be greater than expected and the possibility of participant attrition.

The paired difference between N7FF SRT scores and the participants’ current older SP device SRT scores was calculated. The paired data were compared by a paired t-test to assess the speech recognition difference associated with N7FF against the participants’ current older SP. A minimum significance level of 0.05 was used to establish statistical significance of superiority of N7FF compared to the participants’ older SP.

A paired t test was also used to compare SSQ scores for N7FF vs participants’ current older SP. The SSQ scores were first assessed for noninferiority. We considered the N7FF was non-inferior to participants’ current older SP, if the lower bound of the 95% confidence interval (CI) of the paired difference was higher than the non-inferiority margin of -1. If non-inferiority was achieved, then a superiority analysis was conducted.

#### Follow-up study

As this study was observational in nature there was no formal hypothesis.

Customized questionnaires were designed to capture and quantify attributes specific to the N7 sound processor, that was not available in a standardized tool. Categorical ratings using a 5-point Likert scale (very dissatisfied, dissatisfied, neutral, satisfied, very satisfied) designed to measure the proportion of recipients’ satisfaction over three areas. Hearing performance in specific listening situations (for example, understanding conversation in background noise, understanding the TV); telephone use; and satisfaction and usability of streaming and listening to music with the N7.

Other variables assessed in the customized questionnaires were designed to understand the quantity of participants who used the telephone, accessories and experienced music. The were continuous variables to understand the proportion of participants using features of the N7.

## Results

### Participants

#### Initial study

Forty participants were included in the study; participant characteristics and hearing and device history are presented in Tables 1 and 2.

**Table 1:** Participant characteristics in the initial study.

Parameter		All subjects (n = 40)
Age (years)	n	40
	Mean (SD)	14.5 (9.8)
	Median	11.0
	Min, Max	3, 49
Gender	n	40
	Female	13 (32.5%)
	Male	26 (65.0%)
	Not reported	1 (2.5%)
Participant’s current older sound processor at baseline	n	40
	Nucleus 6/CP900	2 (5%)
	Nucleus 5/CP802 or CP810	8 (20%)
	Freedom	24 (60%)
	Esprit 3G	3 (7.5%)
	Sprint	3 (7.5%)
Speech in quiet (%)	n	40
	Mean (SD)	70.3 (18.05)
	Median	70.0
	Min, Max	32, 100

Max, maximum; Min, minimum; n, number; SD: standard deviation.

**Table 2:** Participant hearing and device history in the initial study.

Parameter		All subjects (n = 40)
Age at onset of initial hearing loss (years)	n	38*
	Mean (SD)	2.4 (7.18)
	Median	0.0
	Min, Max	0, 33
History of hearing loss	n	40
	Congenital with progression	9 (22.5%)
	Congenital without progression	13 (32.5%)
	Progressive	9 (22.5%)
	Unknown	9 (22.5%)
Age at diagnosis of severe high frequency hearing loss (years)	n	39*
	Mean (SD)	2.4 (7.73)
	Median	0.0
	Min, Max	0, 35
Etiology of hearing loss	n	40
	LVAS	4 (10%)
	Genetic	8 (20%)
	Ototoxicity	3 (7.5%)
	Unknown	17 (42.5%)
	Complications at birth	2 (5%)
	Neonatal and other complications	6 (15%)
Age of first cochlear implant surgery in the right ear	n	34
	Mean (SD)	6.9 (8.97)
	Median	3.0
	Min, Max	1, 36
Age of first cochlear implant surgery in the left ear	n	6
	Mean (SD)	6.0 (3.22)
	Median	5.5
	Min, Max	3, 11

LVAS, large vestibular aqueduct syndrome; Max, maximum; Min, minimum; n, number; SD: standard deviation. *Information on all 40 participants not available

Hearing aids were used by most participants with 31 (77.5%) using them continuously prior to their cochlear implant. Most participants continued to use their hearing aid in their non-implanted ear after they received their cochlear implant.

#### Follow-up study

Twenty-nine of the possible 40 participants that were part of the initial Nucleus 7 SP study were included in the follow up study; a total of 5 adults (age range 22–28 years) and 24 children (age range 5–17 years) were recruited (overall mean age 12.8 years) (Table 3). All participants had cochlear implants in one ear only and all participants were using their Nucleus 7 SP for 6 months or longer.

**Table 3:** Participant characteristics in the follow up study.

Parameter		Adults (n = 5)	Children (n = 24)
Age (years)	Mean (SD)	25.8 (2.7)	10.1 (3.4)
	Min, Max	22, 28	5, 17
Gender	Female	1 (3.4%)	10 (34.5%)
	Male	4 (13.8%)	15 (51.7%)
Hearing impairment onset, n (%)	Pre-lingual	2 (6.9%)	16 (55.2%)
	Post-lingual	2 (6.9%)	7 (24.1%)
	Unknown	1 (3.4%)	2 (6.9%)
Hearing impairment duration in years	Mean (SD)	26.1 (1.4)	9.7 (3.4)
	Min, Max	24, 27	5, 16
Participant’s previous Cochlear sound processor	CP810	2 (6.9%)	3 (10.3%)
	CP802	1 (3.4%)	0 (0%)
	Freedom	1 (3.4%)	17 (58.6%)
	Esprit 3G	0 (0%)	2 (6.9%)
	Sprint	1 (3.4%)	2 (6.9%)

Max, maximum; Min, minimum; n, number; SD: standard deviation.

Similar to the initial study, participants were heterogeneous in terms of age, cochlear implant experience, duration of hearing loss consistent with the RWE study design.

### Main results

The raw data collected as part of this study is provided in S2 File.

### Initial study

**Fig 1:**
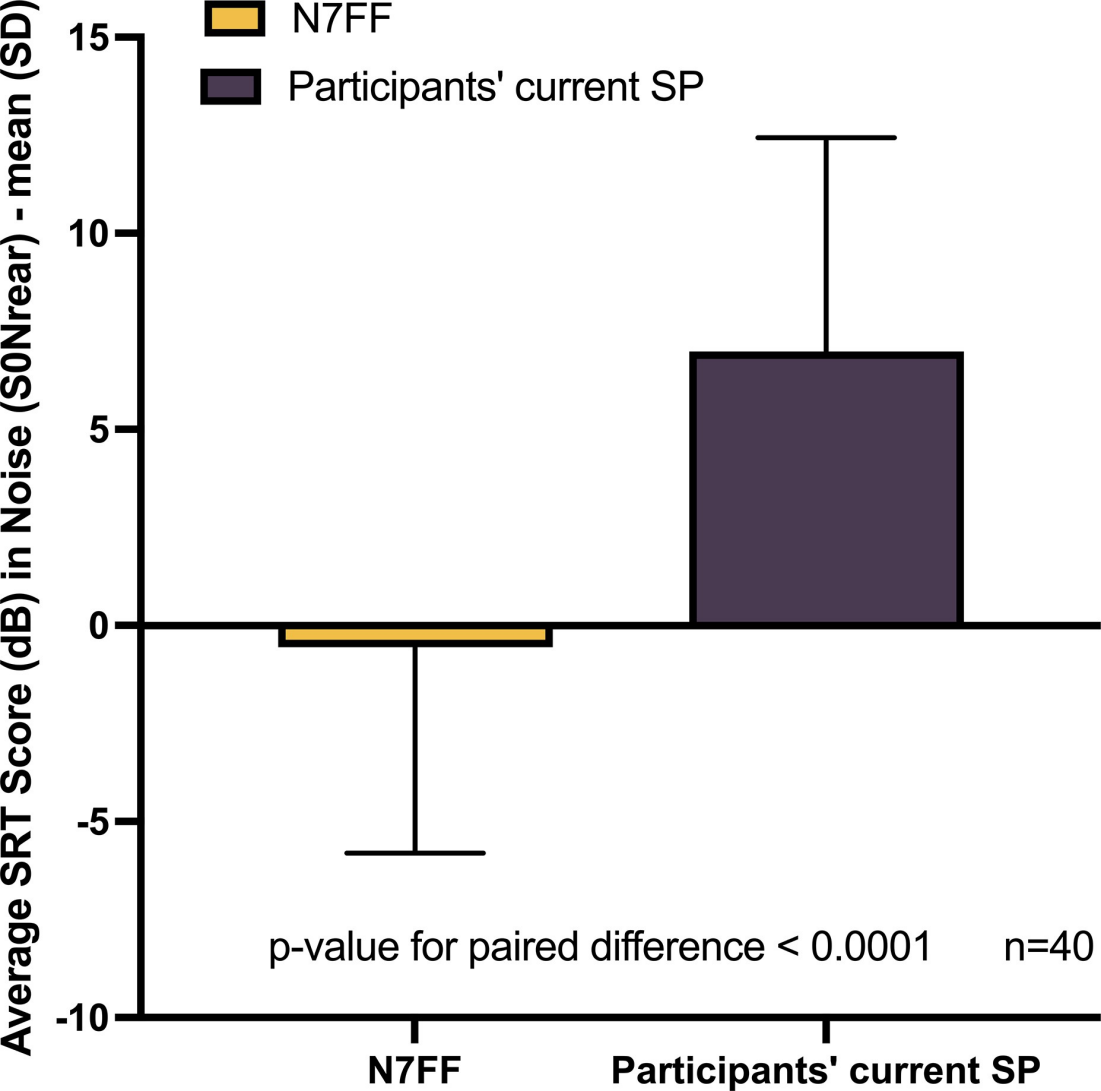
Speech recognition threshold (SRT) score (dB) in noise for Nucleus 7 (N7FF) vs participants’ current older sound processor.

Nucleus 7 SP significantly improved participants’ speech recognition performance when compared with the participants’ current older SP (with their current preferred program), in spatially separated multi-talker babble dynamic noise, with a group mean SRT improvement of 7.54 dB (95% CI 8.49 to 6.60, p-value <0.0001), Fig 1.

**Fig 2:**
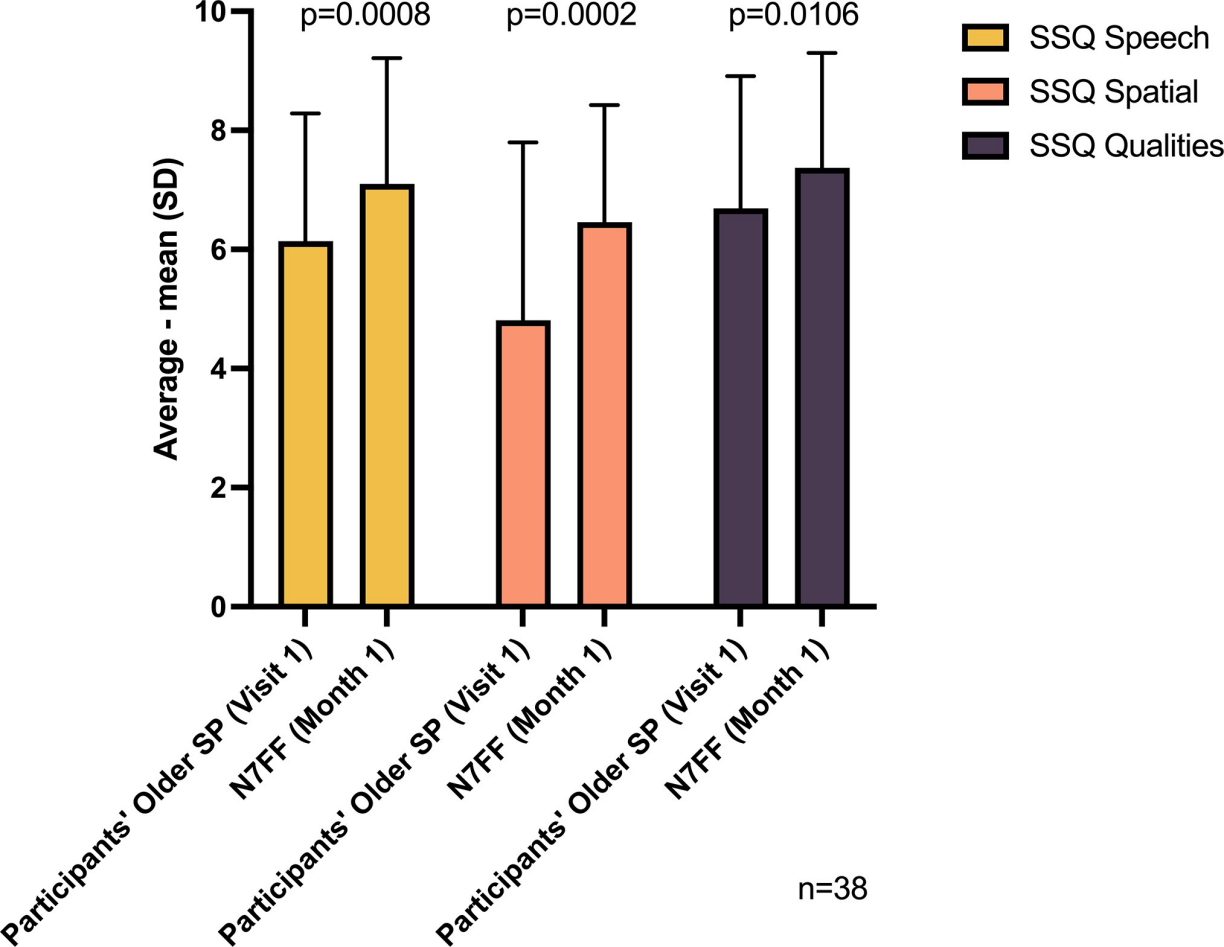
Summary of speech, spatial, and qualities (SSQ-12 and SSQ-P).

Nucleus 7 SP was non-inferior to the participants’ current older SP (with their current preferred program), in terms of the SSQ questionnaire (Fig 2). We also found that N7FF was superior to participants’ current older SP in terms of SSQ. Nucleus 7 SP provided significant improvement for cochlear implant users in their daily life listening environments; the p-values for each of the three SSQ domains all being <0.05.

### Follow up study

*Part 1*: *Hearing performance*. The scenarios within Part 1 of the questionnaire asked the participant to rate their hearing ability in simple one-on-one quiet conversation, small groups in quiet to more complex situations, understanding conversation in noise and localizing alarms/sirens, and finally their overall hearing performance.

**Fig 3:**
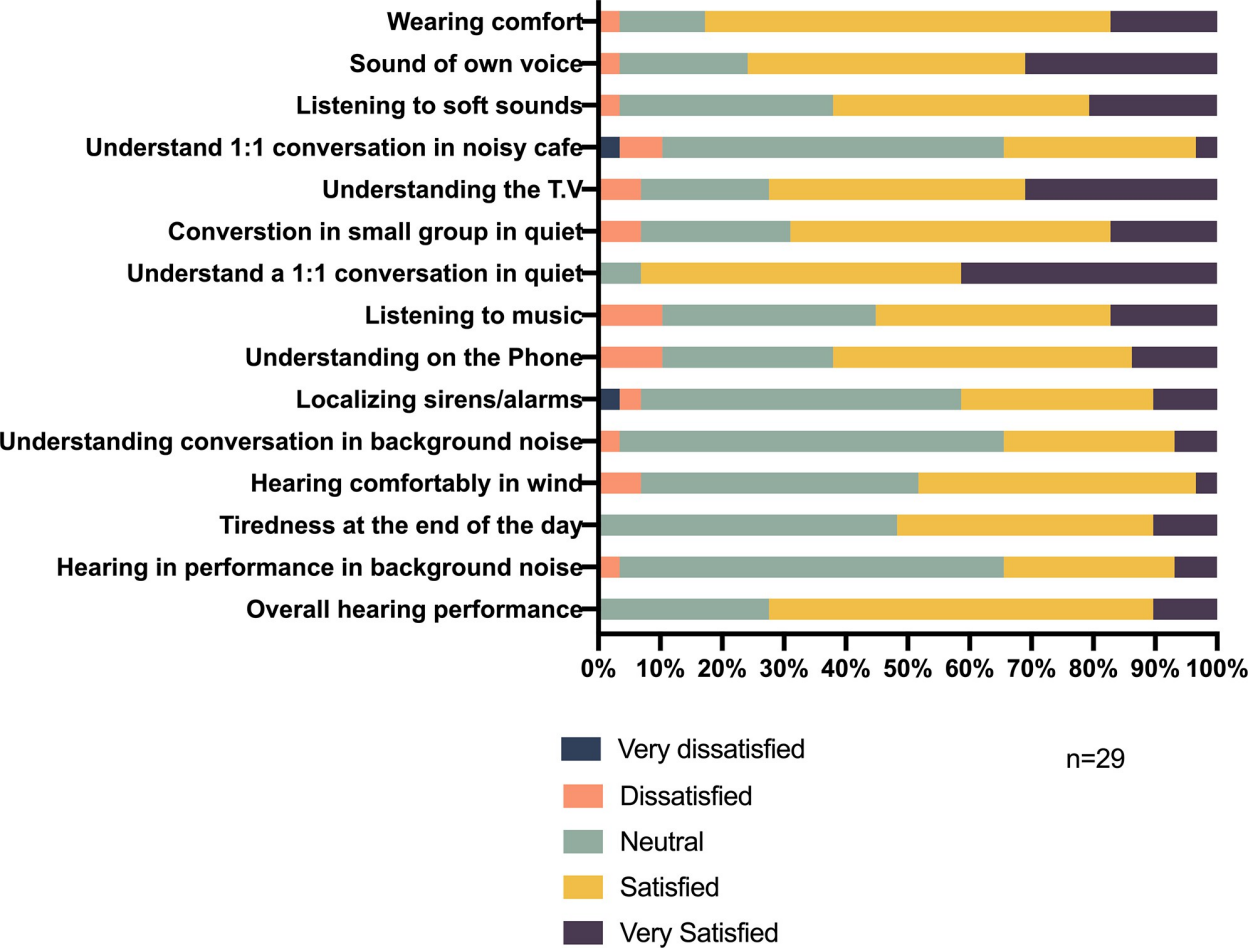
Hearing performance in adults and children combined.

Overall, the majority (72.3%) of participants indicated they were satisfied/very satisfied with their hearing ability when using the Nucleus 7 SP, 27.6% gave a rating of neutral, and no participants were dissatisfied. Satisfaction in different hearing contexts ranged from 62.1% for understanding on the phone, to 69.0% for conversation in a small group in quiet and 93.1% for understanding a one-on-one conversation in quiet situations. Approximately 34.5% of participants felt satisfied/very satisfied in more complex noisy situations, such as hearing/understanding conversations in background noise or a noisy café (Fig 3). Of the 15 questions in this part, very few of the patients were dissatisfied with their hearing performance across the scenarios presented (3.4 to 10.5%). Only two individual ratings of dissatisfaction were given regarding localizing sirens and alarms and one to one communication in a noisy café. This rating was provided by the parents of pediatric participants.

**Fig 4:**
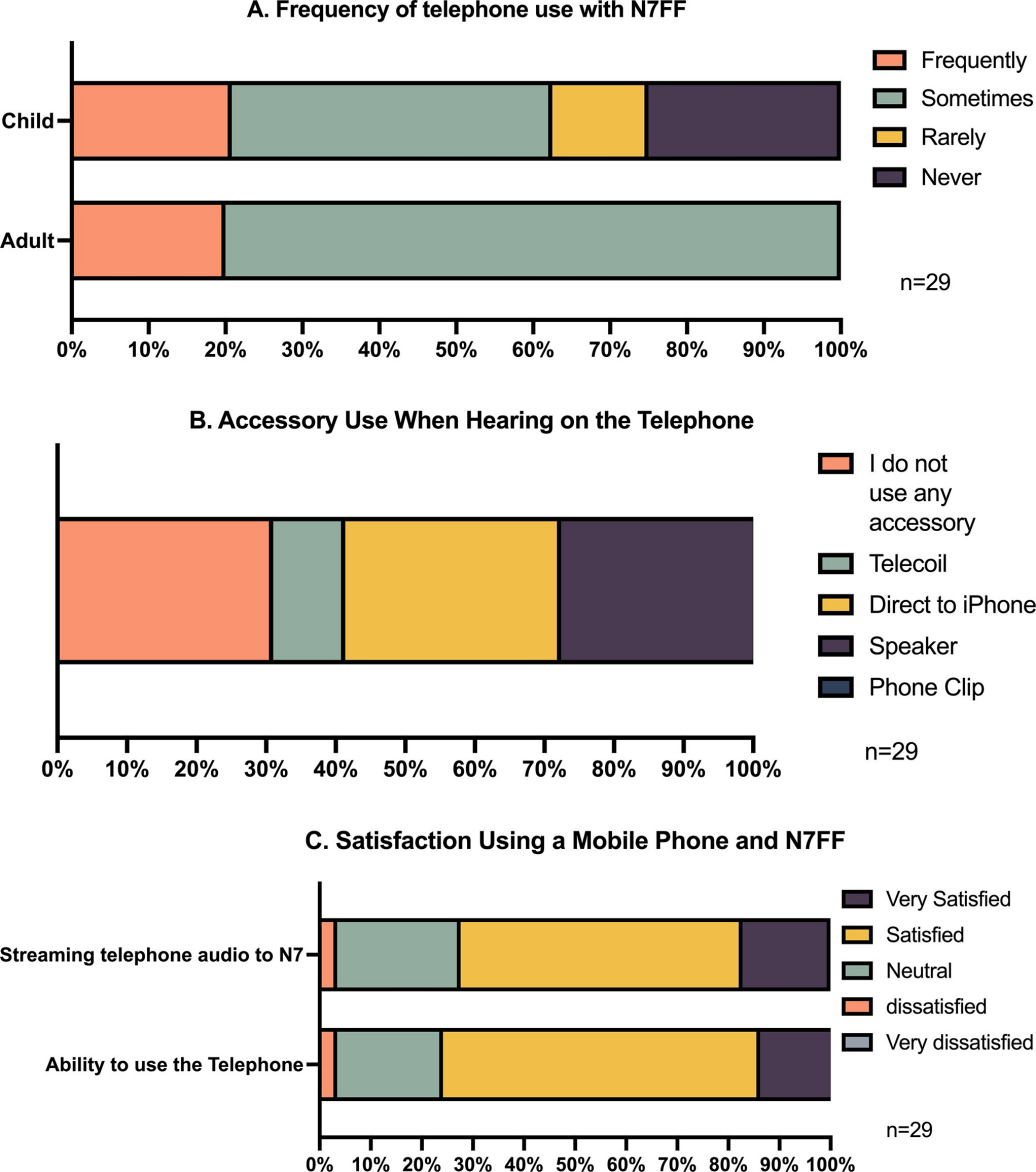
Telephone usability and satisfaction with the Nucleus 7 in adults and children combined.

*Part 2*: *Functionality—telephone use*. Twenty-three (79.3%) participants used their Nucleus 7 SP with the telephone. The frequency of telephone use with the Nucleus 7 SP varied between adults and children; adults were more likely to use the telephone than children (Fig 4A). The participants were not required to use direct streaming or Bluetooth when using the telephone, it was the participants’ decision whether to use this functionality.

Approximately, one third of participants streamed telephone audio directly; others used a smart device on loudspeaker or a telecoil (Fig 4B).

The majority of participants reported telephone use and streaming telephone audio with their Nucleus 7 SP as satisfactory/very satisfactory (75.9% and 72.4%), respectively (Fig 4C). One participant (3.4%) reported dissatisfaction with telephone use and streaming telephone audio. The remaining participants (20.7% and 24.1%) reported a response of neutral for telephone use and streaming telephone audio, respectively.

**Fig 5:**
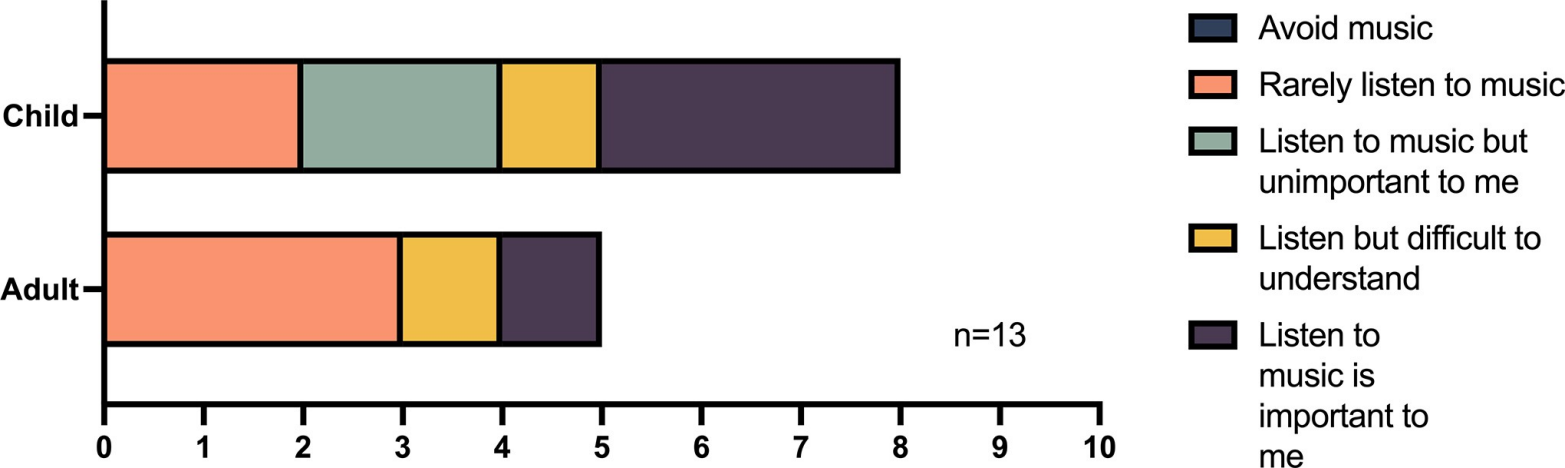
Description of participants’ experience hearing music with the Nucleus 7.

*Part 3*: *Functionality—music and audio streaming*. Thirteen (44.8%) of the 29 participants had experience streaming music to their Nucleus 7 SP as shown in Fig 5.

**Fig 6:**
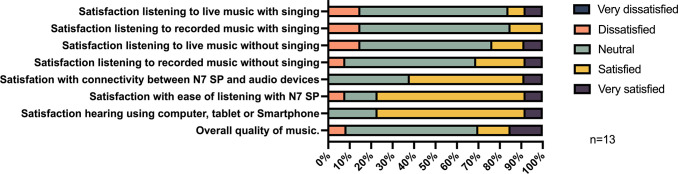
Music streaming usability and satisfaction using Nucleus 7 adult and children combined.

Satisfaction with streaming music/audio directly to the Nucleus 7 SP was rated highly with 76.9% feeling satisfied/very satisfied, 3 participants selected neutral, and no participant selected dissatisfied or very dissatisfied (Fig 6). The connectivity of the Nucleus 7 SP to the streamed audio was assessed in 13 participants and was rated as satisfactory/very satisfactory by 61.5% (8) participants, 38.5% (5) were neutral, and no participants rated dissatisfied or very dissatisfied.

**Fig 7:**
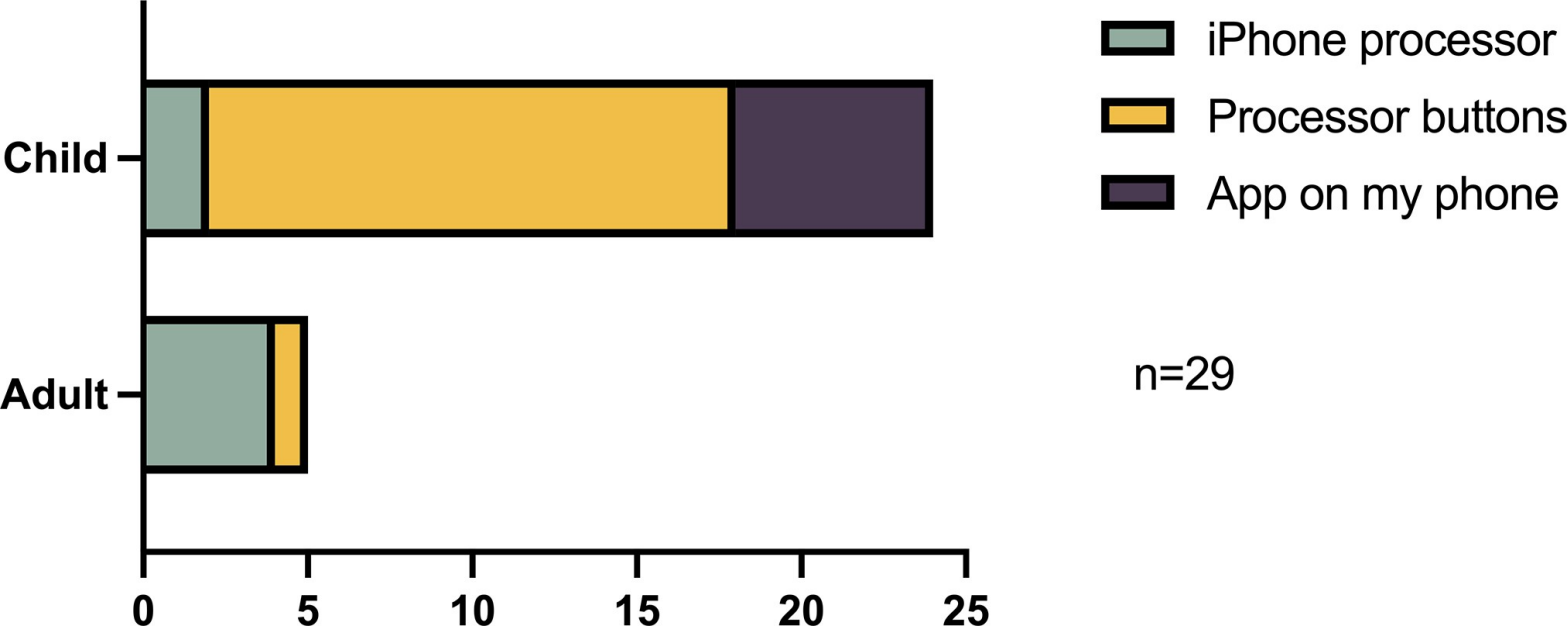
Controlling and monitoring the N7.

*Part 4*: *Functionality of the N7 (Nucleus Smart App (NSA) and N7 features)*. ***Controlling and monitoring the N7*.** The Nucleus Smart App was used by parents of 6 of the children participating in the study to adjust and monitor their child’s device. Adjustments were made using in-built control features linked to the phone (not using the NSA) or the controls on the sound processor by 6 participants (4 adults and 2 children). The majority of participants (n = 17; 1 adult, 16 children) used the controls on the device. The method typically used to control the Nucleus 7 SP varied between adults and children, adults were more likely to use their mobile phone to control their device, whereas children reported using the buttons on the SP (Fig 7). The majority reported being satisfied/very satisfied (51.7%) with the controlling or monitoring of the Nucleus 7 SP with the Nucleus Smart App. Not applicable was selected by 10 (34.5%) participants.

### Features of the N7 sound processor

Participants were asked to list their top 3 features of the Nucleus 7 SP. Each of the features were given equal rating in the analysis. Only 24 of the 29 participants responded to this section of the questionnaire. Of the 24 participants who provided a response, 15 responders nominated 3 features, 7 responders nominated 2 features, and 2 responders nominated 1 feature only. The top 3 features listed by the participants or the participant’s parents were 1) sound clarity and quality and ease of use; 2) size and comfort; and 3) better hearing in noise.

### Safety

There were no adverse events reported during the initial or the follow up study.

## Discussion

In the real world, speech recognition nearly always involves listening in background noise. Such spatially separated listening environments include classrooms, sports settings, restaurants, and family or social gatherings.

Comparative studies between newer SPs and legacy devices are important in order to determine the real-world clinical and user benefit of technological advancements of sound processors for cochlear implant devices [9]. Our study findings demonstrate that Nucleus 7 SP with ForwardFocus provided superior speech recognition benefit compared to the older Cochlear sound processors. In addition, the result of the SSQ questionnaire indicated that Nucleus 7 SP provided a substantial improvement to cochlear implant users in their daily life listening environments, which can be attributed to the utilization of the signal processing technology and the Nucleus 7 Smart App.

The follow-up study demonstrated the hearing ability, usability, and satisfaction of the Nucleus 7 SP in participant’s real-world environments. The quality of the signal by the cochlear implant system is not the only important factor for many cochlear implant users; being able to have a conversation in noisy environments, hear on the telephone, and music appreciation are aspects of hearing that are well known to be challenging for cochlear implant users [9]. There was a high self-reported hearing ability and satisfaction for participants in quiet and complex noisy hearing situations, streaming music, and audio. Furthermore, adults and children reported high satisfaction and usability with several aspects of the Nucleus 7 SP: sound clarity and quality, ease of use and size and comfort.

These findings have important implications for the availability and use of Nucleus 7 SP with Bluetooth connectivity in a Chinese setting. During their schooling, Chinese children spend most of their days in busy, often noisy classrooms, with up to 40 children in each class. In addition, they often participate in extracurricular activities, such as playing sport or playing musical instruments. These environments are generally noisy, and provide a significant challenge for cochlear implant users [5]. However, the superior speech recognition with Nucleus 7 SP could positively impact children’s performance in these dynamic settings. Most children in China over the age of 12 have access to a smart phone, and children younger than 12 often have access to a smartwatch. Typically, in China, children learn foreign languages via Bluetooth connectivity, as such the utilization of the Bluetooth connectivity of Nucleus 7 SP may offer important advantages of the upgraded streaming capabilities offered by the incorporation of the wireless links that is not available with older sound processors [9]. Furthermore, improved speech recognition could contribute to greater socialization and more fulfilling family interactions, as children would not be limited by restricted speech recognition in busy settings such as restaurants or cafes, or large family gatherings. Ability to listen to and enjoy music could also be important to some users and could positively influence well-being [17]. Overall, improved speech recognition, usability and Bluetooth connectivity can help ensure that children with cochlear implants are not disadvantaged compared to their peers in terms of academic and extracurricular performance, and social interactions.

The questionnaire used in the study was specifically developed for use in this real-world study. An important limitation of the study was that a validated questionnaire was not used. Specific to the features of the products a validated measure of hearing ability, such as the 60-item Nijmegen Cochlear Implantation Questionnaire may have allowed comparisons between results of the present study with those of other studies [18]. However, unlike previous upgrade studies with N7, this study was an observational RWE study aiming to identify satisfaction and usability specific to the study device [19]. Additionally, inclusion of such a standardized tool would require participants to complete a total of almost 100 items, which is why a shorter custom questionnaire was developed.

A small number of participants indicated dissatisfied or very dissatisfied with some items on the questionnaire. This could indicate that the quality of the signal provided by the cochlear implant system is not the only important aspect for users [9]. It is possible that some participants struggled to adjust to the use of the new features on their new device, particularly when change in environment was involved e.g. going from a quiet to a noisy space. More detailed recipient counselling focusing on product features functionality and usability to encourage users to access and experiment with the technology in a range of hearing scenes to broaden their experience and understanding of their new hearing device is recommended. This will allow users to optimize their outcomes and satisfaction.

Some participants received assistance with fine-tuning during the study whilst others did not, based on their expressed need and local restrictions due to the Covid-19 pandemic. This could have affected the user experience and possibly impacted the results of the study. However, the impact if any, would have likely been positive as greater fine tuning would have likely led to improved user experience.

The N7 SP can have its volume adjusted with a smart phone via its Bluetooth connectivity, which does not rely on the NSA. However, this functionality is limited to just volume, hence most adults were using it, but only 6 of the pediatric participants were using the NSA. This is likely due to the fact that the Android compatibility with the N7 NSA was not available in China at the time of the study and very few of the participants had access to an iPhone.

In addition, the rating of ‘very dissatisfied’ with regard to localizing sirens or alarms by the parents of the participants, needs to consider that being able to effectively localize and manage in environments with high background noise requires the ability to hear in both ears. Despite the reduction in impact of background noise with N7FF, the device cannot provide the interaural time differences required for effective localization. In such situations, users may need to reposition themselves to maximize the benefits of N7FF.

Furthermore, the relatively high number of neutral ratings for hearing performance may be due to the fact that the questionnaire relied on parents reporting on their children’s experience, rather than the direct reporting of the child’s personal experience. Development of a specified tool that could reflect pediatric use and experience could be warranted for future research.

Through combining performance data from the initial study, and the satisfaction and usability data from the follow up study, it is anticipated these results could guide hearing health care professionals when counselling CI users or caregivers regarding potential benefits of sound processor upgrades. The real-world data collected identified key areas where benefits and satisfaction were clearly measured, and areas of neutrality in user preference. Usability and satisfaction data provided a real-world insight that could ensure realistic expectations of sound processor upgrade and assist in supporting CI recipients in transitioning to new technology.

## Conclusion

The study demonstrated the real-world benefits of using the N7 SP compared with participants older SPs across different hearing environments in a Chinese population and showed improved hearing ability, usability, and satisfaction in participants’ real-world environment. These benefits could have far-reaching effects on academic performance, socialization, and contribute to overall well-being in children and adults.

## Supporting information

S1 File Speech, spatial, and qualities of hearing scale–Questionnaire. (PDF)

S2 File Raw data. (CSV)
