# Supplementary material for: A real-world evaluation of the clinical benefits of improved sound processor technology among Chinese cochlear implant users: A focus on Cochlear Nucleus 7
Source: PLoS One. 2024 Sep 3;19(9):e0307044. doi: 10.1371/journal.pone.0307044 (PMC11371209; doi:10.1371/journal.pone.0307044)
Supplement: S1 File — (PDF) [file pone.0307044.s001.pdf]

Who is completing this questionnaire?	○ Adult	○ Adult on behalf of child

PART 1 Hearing Performance with N7

	Very Dissatisfied	*Dissatisfied*	*Neutral*	*Satisfied*	*Very Satisfied*
Q1. How satisfied are you with your overall hearing performance?	1	2	3	4	5
Q2. How satisfied are you or your child with your hearing performance in background noise?	1	2	3	4	5
Q3. How satisfied are you with your tiredness at the end of the day?	1	2	3	4	5

Please rate you or your child's satisfaction with the following when using the N7 sound processor(s):

	Very Dissatisfied	*Dissatisfied*	*Neutral*	*Satisfied*	*Very Satisfied*
Q4. Your (or your child's) ability to hear comfortably when it's windy.	1	2	3	4	5
Q5. Your (or your child's) ability to understand conversations in background noise.	1	2	3	4	5
Q6. Your (or your child's) ability to know the direction of sirens/alarms.	1	2	3	4	5
Q7. Your (or your child's) ability to understand people on the phone.	1	2	3	4	5

Q8. Your (or your child's) ability to listen to and appreciate music.	1	2	3	4	5
Q9. Your (or your child's) ability to understand a person in a one-on-one conversation in quiet.	1	2	3	4	5
Q10. Your (or your child's) ability to understand conversations with a small group of people in quiet.	1	2	3	4	5
Q11. Your (or your child's) ability to understand what is said on television.	1	2	3	4	5
Q12. Your (or your child's) ability to understand conversation with one other person (1:1) inside a busy café in noise .	1	2	3	4	5
Q13. Your (or your child's) ability to hear soft sounds like birds.	1	2	3	4	5
Q14. The sound of your (or your child's) own voice.	1	2	3	4	5
Q15. How satisfied are you (or your child) with the comfort of the N7 sound processor(s)?	1	2	3	4	5
Q16. Have you (or your child) increased your listening activities since using the N7 processor?		Yes		No	

PART 2 - Streaming to N7

Q17 Do you use a mobile phone or iPad to stream audio to your processor?	○ Yes	○ No		
Q18. What type of telephone do you or your child use most?	○ Landline phone	○ Cellular / mobile phone	○ iPad	○ Do not use the phone
Q19. If you or your child do not use the phone, please let us know why.	______________________			
Q20. What accessory do you or your child use most to connect the N7 sound processor to your preferred telephone format? Select one:	○ Telecoil<br>○ Speaker<br>○ Phone Clip<br>○ Direct to iPhone (Made for iPhone)<br>○ I do not use any accessory			
Q21. How often do you or your child use the telephone to talk with your own sound processor(s)? Select one:	○ Frequently<br>○ Sometimes<br>○ Rarely or if absolutely necessary<br>○ Never			

	Very Dissatisfied	*Dissatisfied*	*Neutral*	*Satisfied*	*Very Satisfied*
Q22. How satisfied are you with your (or your child's) ability to use the telephone?	1	2	3	4	5
Q23. How satisfied are you overall when streaming telephone audio to the N7	1	2	3	4	5

PART 3 MUSIC - Streaming

Q24. Do you or your child stream music or other audio to your N7 sound processor(s)?	◯ Yes	◯ No – If so, go to Q35
Q25. How do you or your child stream audio to the N7 sound processor(s)? Select one:	◯ via Phone clip<br>◯ via Mini Mic<br>◯ Direct to N7 sound processor(s)<br>◯ via TV Streamer<br>◯ Not applicable	
Q26. What best describes the experience for you (or your child) with the N7 sound processor(s) in your (or your child's) best listening condition?	◯ I avoid music<br>◯ I rarely listen to music<br>◯ I listen to music, but it is unimportant to me<br>◯ I listen to music, but it is difficult to understand<br>◯ I listen to music, and music is important to me	

	Very Dissatisfied	*Dissatisfied*	*Neutral*	*Satisfied*	*Very Satisfied*
Q27. How satisfied are you or your child with your ability to hear and engage when using a computer, tablet or Smart phone?	1	2	3	4	5
Q28. How satisfied are you with the ease of listening with the N7 sound processor(s)?	1	2	3	4	5

Rate your level of satisfaction with listening to music in your (or your child's) best listening condition:

	Very Dissatisfied	*Dissatisfied*	*Neutral*	*Satisfied*	*Very Satisfied*
Q29. Recorded music without singing (eg on car radio or home stereo).	1	2	3	4	5
Q30. Live music without singing.	1	2	3	4	5

Q31. Recorded music with singing (eg on car radio or home stereo).	1	2	3	4	5
Q32. Live music with singing.	1	2	3	4	5
Q33. Overall quality of music.	1	2	3	4	5
Q34. How satisfied are you or your child with the connectivity between the N7 sound processor(s) and audio devices (e.g. iPod player)?	1	2	3	4	5

PART 4 Other

Q35. How do you usually control your processor? Please select the most appropriate answer.	○ Processor buttons		○ CR310 remote	○ APP on my phone	○ iPhone
	Very Dissatisfied	*Dissatisfied*	*Neutral*	*Satisfied*	*Very Satisfied*
Q36. How satisfied are you with your ability to control/monitor the N7 sound processor(s) for you or your child with the Nucleus Smart App? If you do not use the App to control your N7 mark not applicable.	1	2	3	4	5
			○ NOT APPLICABLE		
Q37. What do you or your child consider to be the top 3 features of Nucleus 7?	1. ______________________________				
	2. ______________________________				
	3. ______________________________				
